# Supplementary material for: The breakdown of both strange metal and superconducting states at a pressure-induced quantum critical point in iron-pnictide superconductors
Source: Nat Commun. 2023 May 30;14:3116. doi: 10.1038/s41467-023-38763-4 (PMC10229619; doi:10.1038/s41467-023-38763-4)
Supplement: Supplementary file 1 — Supplementary Information [file 41467_2023_38763_MOESM1_ESM.pdf]

Supplementary Information for "The breakdown of both strange metal and superconducting states at a pressure-induced quantum critical point in iron-pnictide superconductors"

Shu Cai[1], Jinyu Zhao[1,2], Ni Ni[3,4], Jing Guo[1,5], Run Yang[1], Pengyu Wang[1,2], Jinyu Han[1,2], Sijin Long[1,2], Yazhou Zhou[1], Qi Wu[1], Xianggang Qiu[1,2,5], Tao Xiang[1,2], Robert J Cava[3], and Liling Sun[1,2,5]†

[1]*Institute of Physics, Chinese Academy of Sciences, Beijing 100190, China*

[2]*University of Chinese Academy of Sciences, Beijing 100190, China*

[3]*Department of Chemistry, Princeton University, Princeton, New Jersey 08544, USA*

[4]*Department of Physics and Astronomy, UCLA, Los Angeles, CA 90095, USA*

[5]*Songshan Lake Materials Laboratory, Dongguan, Guangdong 523808, China*

Correspondence and requests for materials should be addressed to L.S. (llsun@iphy.ac.cn)

Extended data

(1) Temperature dependence of resistance at different pressures and corresponding fits for the sample #2

We performed the same high-pressure measurements on the 1048 superconductor (here we defined it as the sample #2) that was cut from different batches. As shown in Fig. S1, the resistance as a function of temperature for the sample #2 exhibits a similar behavior to that of the sample #1 (Fig.2).

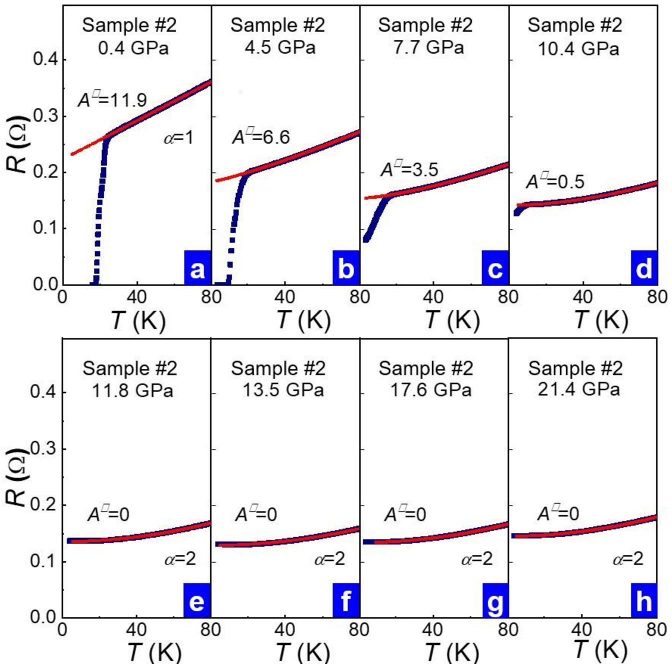

Fig.S1 High-pressure resistance as a function of temperature at different pressures for the sample #2 and corresponding fits by the form of $R = R_0' + A'T + B'T^2$ below the quantum critical point (a-d) and above quantum critical point (e-h).

(2) Temperature dependence of α below the critical pressure (quantum critical point)

The temperature dependence of α is plotted through a logarithmic derivative ($\alpha = d\log(R-R_0)/d\log T$) for the sample #1 and #4 (Fig.S2). It is seen that, the temperature dependence of α does not display a typical quantum critical fan below the critical pressure (quantum critical point), supporting that the strange metal state exists in the ambient-pressure 1048 superconductor.

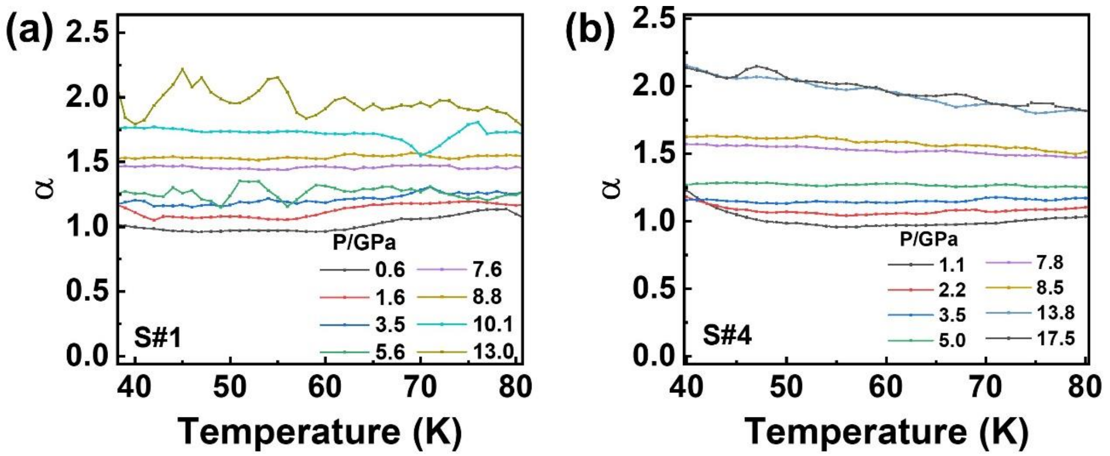

Fig.S2 The temperature dependence of α for the sample #1 (a) and #4 (b), which are surrounded by the NaCl and glycerin media respectively, at different pressures.

(3) High pressure measurements on the $Sr_{0.74}Na_{0.26}Fe_2As_2$ superconductor

To investigate the universality of the correlation between superconductivity and *T*-linear resistivity SM normal state, we carried out high-pressure studies on the $Sr_{0.74}Na_{0.26}Fe_2As_2$ superconductor. We find that, when the pressure reaches 5.9 GPa and 6.6 GPa, a zero-resistivity state appears and its normal state displays a good *T*-linear resistivity behavior (Fig.S3a). Upon further increasing pressure to 10.4 GPa, superconductivity is completely suppressed, meanwhile, its $A^{\square}$ goes to zero (Fig.S3b and S3c), indicating that application of pressure induces the concurrent breakdown of the SM normal state and SC state for the $Sr_{0.74}Na_{0.26}Fe_2As_2$ superconductor. We also find that its T_c and $A^{\square}$ obey the genetic relation of $T_c \sim A^{\square}$ (see Fig.4b).

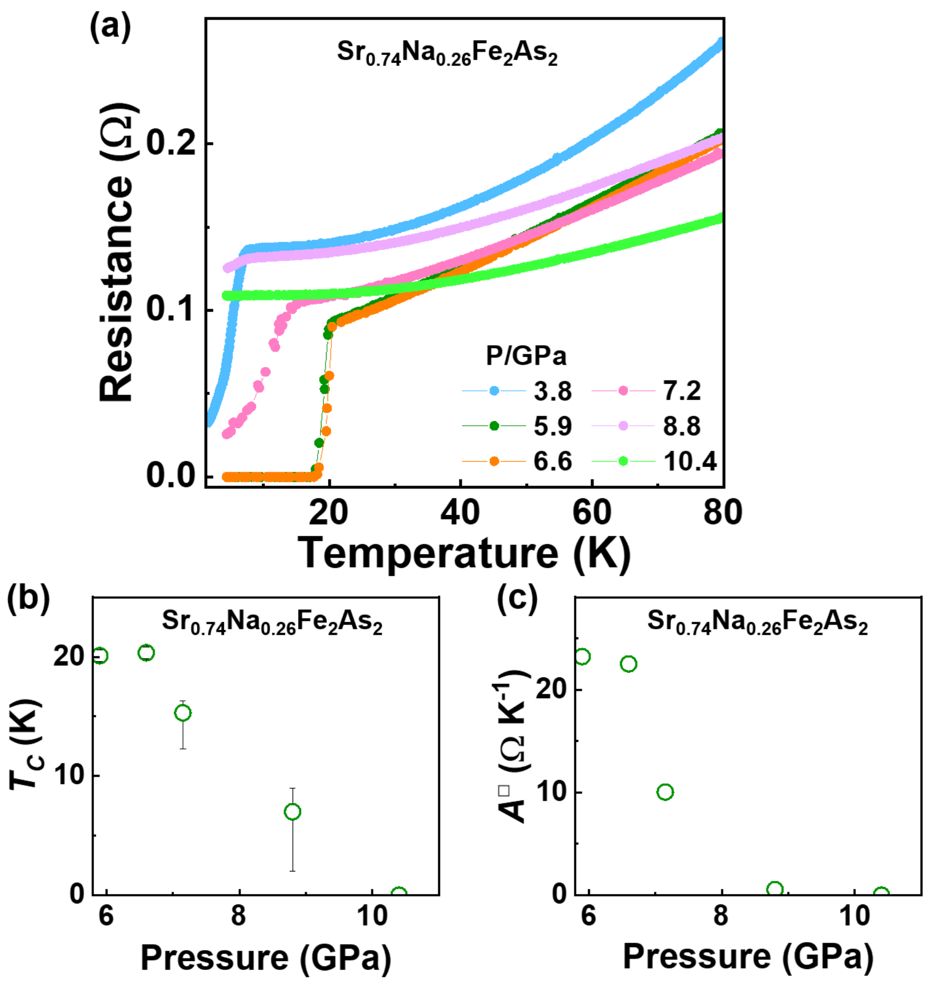

Fig.S3 High pressure properties of $Sr_{0.74}Na_{0.26}Fe_2As_2$ superconductor. (a) Plot of resistance versus temperature measured in the pressure range of 3.8 GPa-10.4 GPa. (b) and (c) Pressure dependence of T_c and $A^{\square}$, displaying that T_c and $A^{\square}$ go to zero together at 10.4 GPa. The error bars represent s.d.
